# Using digital platforms to promote a healthcare provider community of practice for abortion care in Uganda

**DOI:** 10.1186/s12978-023-01721-w

**Published:** 2023-12-06

**Authors:** Dan Kabonge Kaye, Simon Peter Kayondo, Stella Lovina Nabatanzi, Susan Nassuuna, Othiniel Musana, Imelda Namagembe, John Paul Nsanja, Othman Kakaire, Peter Ssebadduka, Cissy Ssekimpi

**Affiliations:** Association of Obstetricians and Gynecologists of Uganda, P.O. Box 11966, Kampala, Uganda

**Keywords:** Digital health platforms, abortion harm and risk reduction, Values clarification and attitude transformation, Community of practice, COM-B model

## Abstract

**Background:**

A community of practice (CoP) is defined as a group of people who share a concern, set of problems, or a passion about a topic, and who deepen their knowledge and expertise by interacting on an ongoing basis. The paper presents a case study on the design, implementation and management of a CoP. The objective is to share experiences, opportunities, challenges and lessons learnt in using digital platforms for clinical mentorships to establish a CoP that promotes enhanced service provision of abortion care.

**Methods:**

We employed competence-based training and ongoing virtual mentorship for abortion care, employing the abortion harm reduction model, and using several digital platforms to create and nurture community of practice for abortion care. Using the Capability-Opportunity-Motivation for Behavior (COM-B) model and textual data analysis, we evaluated the performance of the CoP as a tool to support abortion care, using data from in-depth interviews and information shared on the platforms. The data was analyzed by thematic analysis using text data analytical approach.

**Results:**

CoPs have much unrealized potential for networking to improve abortion care, as they are more inclusive, interactive and equalizing than typical webinars, yet less expensive and can complement (though not replace) physical mentorships. CoPs’ focus on sharing best practices and creating new knowledge to advance professional practice, faces challenges of maintaining regular interaction on an ongoing basis. CoP members need to share a passion for their practice and mutual trust is key to success.

**Conclusion:**

Though it faced initial challenges of connectivity, and limited interaction, the CoP approach using digital platforms promoted shared experiences, personal connections, communication, collaboration and application of knowledge for improved abortion care.

## Background

Knowledge management is a key strategy to ensure implementing into policy and practice what has been learnt [1], and one way to achieve this is through a Community of Practice (CoP). Wenger [2] defined a CoP as *“… groups of people who share a concern, a set of problems or a passion about a topic and who deepen their knowledge and expertise in this area by interacting on an ongoing basis… These people don’t necessarily work together on a day-to-day basis, but they get together because they find value in their interactions, as they spend time together, they typically share information, insight, and advice…”* CoPs are particularly effective where it is vital to ensure the transfer of knowledge into practice, stressing the need for both a shared domain of expertise and repertoire of practices, as well as existence of a sense of community and of interactions that are meaningful so as to delineate identity [2–6]. CoPs offer opportunity for trainees and service providers to connect, communicate, collaborate, coordinate and cooperate during performance after the training [7]. Online mentoring is very useful for professional coaching [8], facilitating interest and choice of career [9] nurturing positive attitudes [10] facilitating resolution of constraints related to geographical remoteness and labor hours [11]. The opportunity for continuous communication and follow-up between mentors and midwives enhances improvement of hard and soft professional skills of the midwives, self-confidence, transition from novice to expert, and empowerment [11].

Establishing and sustaining COPs in organizations is a challenging endeavour and COPs are likely to face various challenges and difficulties throughout their life-cycle [12–14]. As CoPs are promoted in healthcare as a tool *“*to enhance knowledge and improve practice”, they provide a means for knowledge transfer across boundaries, to generate and manage a body of knowledge for members to draw on, or to promote standardisation of practice, through innovations in ideas, knowledge, and practices [12]. Wenger et al. [13] underscored the need for organisations to actively and systematically cultivate CoPs for their benefits. In line of this, if CoPs are to be cultivated, there is a need to scrutinize how functional they are and to assess their impact in improving healthcare practice [4], yet documented information on this aspect is lacking [4]. The growing movement towards, and the growing demand for competence-based training and quality improvement highlight the need to understand how students learn or how workers incorporate the competences (knowledge, attitudes, skills and decision-making) into routine performance during practice. This more relevant considering that integrating research evidence into practice is that it involves a complex process of acquiring, converting, and applying a mix of explicit and tacit knowledge in clinical activities [4].

There is limited data on organisational processes, experiences, perceived effectiveness and challenges related to success of CoPs in healthcare. Despite the growth of CoPs in health settings, there are few publications on how they work and support health programmes or the challenges they face [4, 12–14]. Though they are complex social phenomena, CoPs can be studied through a realist lens, where members and organisations are embedded in multiple social systems, as the different expectations and experiences of members influences outcomes of the CoP [12]. In this lens, the outcomes might subsequently change the members’ experience ad expectations, and this might change the context and conditions that made the CoP work in the first place [12]. Outcomes of the CoP are affected by different contexts on different levels (individual, interpersonal, organisational and contextual). The objective of the paper is to share experiences, opportunities, challenges and lessons learnt in using digital platforms for clinical mentorships and the abortion harm reduction model to build a CoP that promotes connection, communication and collaboration for enhanced service provision in comprehensive abortion care.

CoPs have potential to improve quality of care through better knowledge sharing in several areas [15], which include review of feedback reports, collaborative improvement meetings, real-time communications, sharing challenges, exchanging knowledge, engagement in group problem solving, and enhanced social support. CoP engagements foster a shared sense of ownership of the CoP, better motivation to suggest and implement them to propose and carry out innovations, and strengthened social and professional identities within and outside the group, in addition to improved self-efficacy [15], as well as better group learning and enhanced problem solving [16, 17] as well as improved work-related knowledge, confidence, performance; social capital and social status [18–21]. Dunn et al. [22] present a case study of how a virtual CoP was used to address barriers and facilitators for introduction of mifepristone for medical abortion [22]. The sustained engagement enhanced knowledge, improved practice, supported innovation, addressed implementation barriers in real time, improved ability and willingness to initiate medical abortion, and facilitated adoption of medical abortion as a practice among the participating clinicians [22].

### The theoretical frameworks

#### Abortion harm reduction

Harm reduction is an evidence-based public health and human rights framework that prioritizes strategies to reduce harm and preserve health in situations where policies and practices prohibit, stigmatize or drive human activities (such as abortion care) underground [23–25]. The abortion harm reduction (AHR) model focuses on lessening the harms associated with pregnancy termination in contexts where abortion is restricted [23–25]. In the Ugandan context, abortion is only permitted for the purpose of saving a women’s life, and is not available on request. The comprehensive abortion care (CAC) using the AHR framework emphasized the need to address the harms associated with the whole abortion trajectory (in a restricted abortion context), from unintended pregnancy to provision of postabortion care. The AHR model highlights harms of the context at conception of unintended pregnancy and the root causes, which corresponds to primary prevention of abortion complications), through seeking and provision of termination of pregnancy (which corresponds to secondary prevention, and includes counseling on the option of accepting the pregnancy and continuing with antenatal care). The provision of postabortion care (which corresponds to tertiary prevention) emphasizes emergency management of abortion complications, counseling, postabortion contraception and linkage to other SRH services. Discussions emphasized the abortion laws and regulations and their effect on abortion care. The model was implemented for primary, secondary and tertiary prevention of complications that lead to abortion-related deaths while simultaneously addressing related stigma and discrimination and advancing women’s reproductive health rights. The AHR model has been found acceptable and potentially effective in advocacy for addressing the burden of morbidity and mortality from abortion complications [26, 27].

#### Values clarification and attitude transformation

The Abortion values clarification and attitude transformation (VCAT) framework [28] posits that values play a critical role in determining how people make decisions and ultimately act. In VCAT interventions, facilitators lead audiences through a process in which audiences analyze their personal values, attitudes and actions related to abortion. Audiences engage in honest, open-minded and critical reflection and evaluation of personally-relevant abortion information and situations related to abortion practices or management, including the underlying factors which constitute the root causes of abortion [26, 28]. In this approach, audiences analyze the positive and negative (including harmful) consequences of their beliefs and attitudes towards abortion patients in general and abortion care in particular, and can thereafter progressively align their personal and professional values to be able to meet their professional obligations ad responsibilities [26–28].

#### The COM-B model for behavior change

The COM-B model for behavior change [29] posits that capability (C), opportunity (O), and motivation (M) are three key factors influencing behavior (B). Intervention methods, as interveners need to ensure the sustainability of learned behavior. To engage in a behaviour (B) at any given moment, a person must be physically and psychologically able (C) and have the opportunity (O) to exhibit the behavior, as well as the want or need to demonstrate the behaviour at that moment (M). Capability refers to an individual’s psychological and physical ability to participate in an activity, while opportunity refers to external factors that make enactment of a behavior possible. Motivation refers to the conscious and unconscious cognitive processes that direct and inspire participation. In the context of a CoP, behavior connotes active participation in CoP activities. The COM-B model is particularly important when considering establishing and nurturing a CoP, as it delineates what components of behavior need to be changed in order for a CoP to be successful. Similarly, it outlines the structural elements a COP should provide to members to facilitate and motivate their active engagement. These elements included sharing experiences of challenging cases, asking questions about difficult cases, making phone or video calls with content related to provision of abortion care services, sending or replying to messages, proposing new discussion themes, collecting and submitting data related to abortion care, or participating in the interactive zoom-based CPD seminars. Thus, active participation referred to regular communication, connection, collaboration, sharing experiences and challenges, and showing eagerness for further learning and problem solving as well as putting acquired knowledge and skills into practice.

## Methods

This project was implemented by the Association of Obstetricians and Gynecologists of Uganda (AOGU) to training and mentor midwives, between February 2022 and March 2023. We present a case study on implementing a CoP using various digital platforms. Our concept of the CoP was that of a group of healthcare providers who provide comprehensive abortion care (CAC) using the abortion harm reduction model, and who utilize the CoP as an arena for connection, communication, interaction, collaboration and sharing in relation to provision of abortion care.

The goal of the project was to establish and nurture a CoP in sexual and reproductive healthcare, especially CAC, to build capacity of midwives and doctors as a CoP that supports abortion care services provision according to the 2022 World Health Organization (WHO) guidelines [30]. The current training of midwives does not include competence-based training in postabortion care let alone provision of safe abortion. The project assumptions were that midwife refresher training shall improve provider knowledge, attitudes and skills to support abortion selfcare or provide CAC. Also, competence-based trained midwives, whose attitudes would shift from negative to neutral may be less likely to obstruct (and may even support or facilitate) women’s access to abortion care and colleagues’ postabortion service provision. Similarly, midwives and doctors with positive attitudes may be most likely to provide, support and advocate for safe abortion. Besides, improvements in attitudes and behavioral intentions predict health workers’ willingness to support or provide abortion care services or improve the quality of care they provide, based on value clarification and attitude transformation (VCAT), an improved understanding of women’s right to abortion and consequences of poor access to quality care.

The project strategy was to promotes a change in the abortion narrative through building capacity of doctors and midwives to provide CAC as well as engage in open discussion on abortion that would result in reduction in abortion stigma. The theory of change for the intervention was that competence-based abortion care training (using the AHR model) would provide knowledge and skills and nurture positive attitudes to health workers through use of realistic scenarios, critical self-reflection, sharing empathy-evoking experiences. Dialogue on abortion beliefs, health workers’ values and professional ethics and responsibilities would promote positive attitudes that are supportive of women’s right to safe abortion care and mitigate abortion stigma. The specific objectives were strengthening these health workers’ competence and confidence to support abortion selfcare and PAC using the 2022 WHO guidelines; mitigating abortion stigma through strengthening access to SRH information by the community and health workers via a call centre, online applications and other digital platforms; and strengthening the generation and use of abortion-related data to inform policy and practice.

The project activities involved training 120 health workers (mostly midwives and 12 doctors) in comprehensive abortion care. The training content included the burden of abortion as a cause of maternal morbidity and mortality, the concept of comprehensive abortion care, the abortion harm reduction model, client-centred counselling, client-assessment, management of abortion complications, pain management, referral and linkages in SRH, and techniques for evacuation of the uterus. Other content included VCAT in relation to the AHR model, abortion-related data collection and use and the law/regulatory and policy framework on abortion. The postabortion contraception content included the role of family planning in maternal health, the different methods for family planning, family planning counselling (the REDI framework), family planning and other drug interactions, management of abortion side effects, and the different models for integrating family planning services in clinical practice. The REDI framework (Rapport building, Exploring, Decision-making, and Implementing the decision) [31] offers a comprehensive approach to strengthen counseling for contraception.

AOGU has mentors who provided the training. Training sessions employed didactic lectures, brain storming interactive discussions, group work, role plays and simulation training (using pelvic models for contraception and a both pelvic models and water melon model for uterine evacuation), before trainees had clinical experiences with two days of clinical practicums. The trainees had and performed well on both the pretest/post-test assessment (which assessed mainly knowledge and its application), and on attitude assessment with the VCAT questionnaire, indicating adequate knowledge and positive attitudes for using the AHR model for abortion care among the health workers at the inception of the CoP. The CoP participants were mainly midwives (108) and 12 doctors, all aged between 24 and 48 years, with years of experience ranging from 2 to 24 years.

Training was complemented from 2 weeks later with continuous virtual mentorship continuing professional development (CPD) engagements using virtual digital platforms (a call centre, WhatsApp and other social media and short messaging services (SMS) texts. In addition, we arranged bimonthly updates on the comprehensive abortion care topics, progressively moving from theoretical knowledge to applied practice. The call entre was also expected to provide telehealth mentorship to the trained health workers, thus fostering a CoP in SRH. The digital platforms were meant to provide a forum that promotes ongoing learning through sharing experiences, challenges, opportunities and updates. The trained health workers were provided with a modest remuneration to compensate for their attendance and participate in in the CoP activities. Additionally, for 20 facilities, tablets with a data collection software were procured to strengthen the collection and transmission of real-time abortion-related data, and the trained health workers who participated in this activity received regular remuneration.

### Data collection

#### Data from using the digital platforms

Information was collected from the mentees on their experiences in using the abortion harm reduction model. Data was also collected on user experiences on using various digital platforms (from the discussion groups) such as *Google meet*, WhatsApp texts data the *zoom* presentations, phone calls and SMS texts) used for mentorships, as well as from user experiences in the project reports, and highlighted challenges, barriers and facilitating factors participants identified when using the digital platforms.

#### In-depth interviews

 The Virtual and in-person interviews were conducted by two people who were part of both the initial training and the on-job clinical mentorships. Participants in the interviews were purposefully selected from those health workers earlier trained and connected on the various digital platforms. The issues explored were users’ experiences, challenges, perceived barriers and perceived opportunities for using the various digital platforms to enhance learning and performance in provision of comprehensive abortion care. The data from interviews was collected from 40 respondents (which targeted at least one respondent from each of the 50 health facilities where these respondents worked). The interviews were conducted 3–6 months after the training, that is, after the initiation into the CoP.

#### Textual data

 The responses from the (in-person and virtual interviews) and the summarized discussions on the digital platforms were converted into transcripts that formed text data.

#### Ethical considerations

 All the respondents gave permission for their data and information discussed to be included in both reports to the project funders or publications or dissemination meetings with the assurance that individual information would be anonymized and no person-identifier information (such as names, telephone contact or individual health facility) would be indicated.

### Data analysis

Textual analysis refers to several research methods used to describe, interpret and understand texts including the assumptions, values revealed, symbolisms, and iteral meaning to the subtext [32]. Textual analysis is conducted to illuminate the underlying political, social or cultural context being investigated [32]. In order to understand individuals’ perceptions, you have to understand and analyze the way individuals attribute meaning to what goes on around them, or to find out how they react to action (or lack of action) [32]. Data analysis was done using textual analysis. The data analysis was conducted by two people, however, ii involved thematic analysis of emergent themes. The final themes were agreed upon by consensus, after analysis of the contexts and likely sentiments in which the responses were made or the texts were submitted. Textual data analysis involved the following steps: analyzing spoken or written responses (individuals’ spoken words turned into text), interrogating planned events (what are the motivations, predisposing factors and consequences of their action), identification of patterns and identifying the context of the statement or action, analyzing the variations or exceptions (and generating tentative explanations for the patterns and checking to see if they are present or absent in other settings or situations); working explanations into a theoretical model/framework (linking the commitments to a planned behavior).

From the text data, the emerging factors were aggregated into barriers (difficulties, challenges, encountered problems) and facilitators (any factors that enhanced connection, communication or ongoing collaboration and any factors that improved engagement were classified as facilitators). The later were then grouped in whether they enhanced capability, opportunity or motivation according to the COM-B model.

## Results

In our study, the anticipated behavior was active participation in the CoP activities. *Capability* related to physical and psychological abilities that enable an individual to perform the behavior, such as having the necessary skills and knowledge. Opportunity related to the external factors that make the behavior possible or prompt it, considering environmental context and social influences. *Motivation* related to brain processes, (both reflective and automatic), that strengthen and direct behavior, like intentions, beliefs, emotions, and habits. Thus, enhancing knowledge, skills and decision-making and implementing strategies to improve health worker communication, connection, collaboration and engagement, including via the innovative virtual methods was related to *Capability.* Creating supportive environments with provision of mentorship support, supplies, equipment and updates and facilitating collaborative learning referred to *Opportunity*. Provision of information updates, an opportunity to share challenges and experiences and refund of expenses on virtual connections in the CoP related to *Motivation.*

### Capability

*Capability* related to physical and psychological abilities that enable an individual to perform the behavior, such as having the necessary skills, values/attitudes and knowledge, as evidenced by the pre-and post-training scores. The initial training in CAC and VCAT improve the participants knowledge and improved awareness about the positive values that they required to provide CAC as part of a CoP. In the CoP, the ongoing discussions on knowledge, challenges, opportunities for services improvement and sharing experiences reinforced provider knowledge and improved their individual value clarification and attitude clarification to enable them balance their professional and provider values, address value conflicts and meet provider obligations in provision of CAC in a legally restrictive context.

### Motivation

Efforts to foster a CoP may identify facilitators of a CoP. The task may be easier in achieving the objectives if the efforts build on a prior existent “de facto’ CoP. Unfortunately, for our situation, this was the first time to focus on this kind of activity. There was a lot of enthusiasm expressed from posts by participants on the WhatsApp forum:

*“Am glad to be part of this forum. I hope to share and learn much from my colleagues” This (training )has made our performance better…We are a winning team”. Midwife,IDI*.

*“Thanks for sharing the presentation. I will be very helpful for sharing information during our facility CMEs.” Midwife*.

*“Thanks for adding me on this platform. …I have (been getting) so many questions from patients (for which) I need answers…We expect to learn much and eventually perform to our capacity and to your expectations…We all shall perform better” Midwife*.

*“It is interesting to learn more online…The challenge is the time (making time available to attend the online zoom meetings)…we will make attending a priority, because we have similar problems…We can share solutions or get help from each other”. Midwife*.

*“Thanks for sharing updates…this interaction has greatly improved referral (pathway)…We have the same challenges… discussions apply to all of us” Midwife, IDI*.

Over time, the number of active members on the forum remained about 50 out of the 80 members, despite several reminders by e-mail, WhatsApp, Short messaging service (SMS) text and telephone calls. Some of the reminders were from the midwives themselves to colleagues:

*“Please fellow midwives and colleagues, we need to know more (through interaction). Some of us are “freshers and we are happy for the knowledge we receive”. Midwife, IDI*.

*“I am humbled by the updates…. there is much to share and learn. Even tomorrow, we are ready… let’s work on time management…that is the biggest challenge” Midwife*.

*“We are many on the forum, but few (of us) regularly attend the CMEs…Maybe the timing (of 0830 hours) is wrong (not conducive)… I encourage colleagues to use alarm reminders in their phones…Mine works very well… It (alarm) can wake you up when there is a meeting (if you overslept)” Midwife*.

Exposing trainees, to new knowledge has traditionally been done through formal professional development activities, such as seminars and conferences, often with large numbers of attendees. While there is still clearly value in these forms of teaching and learning, this top/down approach, with internal or external ‘experts’ presenting to a relatively passive and unengaged audience has limitations in ensuring that the either adequate knowledge is acquired or the acquired competences are implemented routinely during performance of the trainees. The CoP ensures that members learn from each other, not necessarily from experts, and emphasizes application of the knowledge as well as positive work-related values, making it easier to relate the new knowledge to the work environment and to improve and sustain performance. Midwives expressed satisfaction with the professional development using digital platforms, as they found the approach flexible, sustainable and complementary to the training received earlier, as noted by the respondents:

*“I would suggest that since this forum is created to address some of the issues we face (at work) and for sharing knowledge, it is better to ask (each other) the questions which clients are asking so that we debate on them and get answers…This group has mentors on it… I feel free to ask any questions. I get all the answers”. Midwife, IDI*.

*“Thanks for creating this group. This group was created for learning purposes…Thanks for emphasis of value clarification and professional values… Doctor, IDI*.

*We are benefitting much (a lot)…this makes learning easy…We have a lot on common…We need to share…We are going to work hand in hand” Midwife, IDI*.

*“Thank you team for attending the CMEs and coming for the digital data collection training updates….We are now a team…We have similar problems and concerns… (Now that (I am seeing you all and (it) seems like I can see all your smiling faces” (Mentor,)*

*“…Your efforts are highly appreciated…because of you and the CMES, I received promotion after my interviews…because of that I have a debt to pay by providing quality services” Midwife*.

### Opportunity

Having a common understanding of what the CoP was and what it was meant to achieve is often a challenge for CoPs. Having a well-articulated design is key. Possession of core of communication and group process skills are critical for facilitator success, yet these were not assessed but were assumed. Technology related issues were prevalent, as not all midwives possessed or regularly used the digital platforms like WhatsApp, and midwife anxiety, systems connectivity and systems connection reliability were a common problem. *Orientation* of mentors and midwives about the goals of the mentorship influences the success of mentoring, through knowledge resources that include the different types of knowledge and expertise held by the members. At times, there were challenges of connectivity, as noted by several midwives:

*“Is it only me with sound issues? I have joined by don’t hear anything.” Midwife*.

*“ I have all along permitted the zoom audio, but (are) not hearing anything” Midwife*

*“The volume (sound) is not clear…Sometimes too loud or there are interruptions…may be presenters should also reduce on the speed…” Midwife, IDI*.

*“Colleagues, thank you all for attending… more interaction is needed…the internet failed just before the end of my presentation. I am happy we had gone far by the time I lost (my) internet (connectivity)”.Mentor*.

The focus on the personal interaction as well as managerial and instrumental aspect has practical value in improving effectiveness of the CoP. Many CoP members got to know each other personally, which markedly improved communication with each other

*“The majority of us were not attending util recently. Is the problem (that is stopping people from attending) smart phones, data, network, (using) technology or timing? …I think it is all these…You see, it is good to highlight all the challenges…… we needed time to know each other…AOGU are good people…They might take us through (using) zoom orientation”. Midwife*.

*“Thanks for the session (The value clarification and attitude clarification session on why the trajectories that lead to abortion deaths). This will shape our professional values. .and thanks for sharing the presentations” Midwife*.

The theory of change needs to be clear to all members to a CoP. To achieve CoP objectives, a more explicit ‘theory of change’, including how to monitor and evaluate effectiveness needs to be explicit. While the objectives and relevance of the CoP were articulated at every opportunity, some midwives seemed not to have grasped them. Some, on several occasions, suggested discussions on pregnancy complications or other medical topics (when requested to choose a topic for discussion):

*“Shouldn’t we choose any topic? What if I choose management of hypertension in pregnancy?… I think we need to cover antepartum hemorrhage” Midwife*.

*Are the discussions only about abortion? What about other problems? Midwife, IDI*

Resources also include access to information collectively and through its members, and any pre-existing knowledge-sharing platforms, as well as the acceptability of different media for communication (such as phone calls, SMS texts, and chats during the zoom presentations and thee WhatsApp written, audio and video messages). This variety is necessary as individuals participate in CoP for diverse reasons, among which are that the CoP directly provides knowledge value, personal connection, and opportunity to improve their skills.

Time resources relate to the time that members choose to allocate to the CoP activities and the time that their organizations allow them to take out of other, more formal activities. While it was challenging to identify a time that was suitable for all. A time of early in the morning (0830–0930 h) was reached by consensus as the most optimal time. The participants greatly appreciated the opportunity to participate in sharing experiences and knowledge across multiple digital platforms, as exemplified by two respondents:

*“We are excited about the opportunity to share updates and challenges with colleagues. The forum has mentors to address all and any questions that we present” Midwife*.

*“Thanks for keeping us updated. Is it possible to share the presentation? … Can our moderators share the main points for those who missed the zoom presentations? Midwife*.

Since CoPs are voluntary, what makes them successful over time is their ability to generate excitement, anticipation, relevance, and value to attract and engage members. Often, trainees and service providers show keen interest, as noted by a midwife:

*“The presentation has been wonderful. I shared the link with my colleagues, who were delighted about this. However, they were unable to log in, but we listened to the zoom slide presentation together… I see this as my personal initiative. I have shared the presentation with them” Midwife*.

Social learning, collaborative learning and thinking together are key concepts in a CoP and what makes it work or fail. Thinking together is conceptualised in the way members share knowledge through mutually guiding each other in the area of shared interest. To achieve this, c*onfidentiality and security* of that shared space are a critical component of successful mentoring relationship. Mentorship was enhanced by establishing personal and professional friendship and relationships through sharing personal stories. Besides, financial and other material resources include funds and in-kind allowances (meeting space, web space, materials, etc.) as noted by midwives:

“*We share the data we receive from AOGU. We also share the slides and materials. Thanks to AOGU for our data refund for the zoom meetings. This is great.” Doctor, IDI*.

*“At my facility, they appreciate the zoom presentation, as I use the slides obtained to share updates during CPD seminars at my workplace” Midwife, IDI*.

Our CoP concept was able to achieve the establishment of an arena for communication, interaction, sharing and problem solving in relation to provision of CAC. The CoP also provide a forum for continuing VCAT, so that members could identify, discuss and analyze vale conflicts between professional and personal values, and eventually and adopt/adapt values and beliefs that are aligned with their professional obligations regarding provision of CAC. The CoP improved both understanding and application of the AHR model for expanding access to CAC, as the AHR emphasizes addressing harms related to abortion, through primary, secondary and tertiary prevention of abortion complications.

## Discussion

Our project describes attempts of using the CoP concept to improve understanding and application of the AHR model for improving access to CAC in a context where abortion is legally restricted. Fostering different opportunities and levels of participation, and emphasis/focus on values are critical for the CoP. In our findings, *Capability* related to physical and psychological abilities that enabled participation in the CoP activities, *Opportunity* related to the external factors that enhanced participation in CoP activities, while *Motivation* related to CoP rmembers’ interpretation of the events, experiences and activities and how these influenced their beliefs, intentions and participation in the CoP.

Our findings present a case study on initiation and operation of a CoP among health workers, with related challenges in both conceptualization and implementation. Designing a CoP to achieve both aliveness and effectiveness is challenging as involves creating structures, systems, and roles for different members, being flexible and tolerant to values of other members, ensuring the knowledge shared is applicable to the members working environment and maintaining direction, character, vibrancy and energy. Being members of a CoP did not indicate having similar views and values in relation to far health workers were ready to implement comprehensive abortion care. While all were knowledgeable about comprehensive abortion care and could identify some aspect of care they could routinely provide along the abortion trajectory (from primary prevention to tertiary complication of abortion complications), few were willing to provide safe abortion using manual vacuum aspiration. The choice of how much they could offer depended on their value clarification. Many were able to align their personal and professional values via VCAT, and thus were able to provide care to the extent of their clarified values, and always provided harm reduction counseling to clients. Therefore, using the AHR model as the bedrock of the CoP strategy-due to its inclusiveness—could be particularly appropriate at bringing together health workers with different views and values onto the same platform.

All CoPs, whether planned or spontaneous, need coordination. Our CoP had a “coordinator” who organized the zoom seminars and mentors stimulated interaction within the WhatsApp group. While it was envisaged that midwives would initiate the discussions, they often did not initially, and even when prompted, few of the midwives participated in the discussions. The six mentors also actively stimulated lively discussions, focused on application of knowledge. Besides, mentors spontaneously took on leadership roles, actively participating in the discussions and debates, and suggesting varied topics which emphasized the application of knowledge and the values/attitudes that promote access to care. Even then, even with prompting by the mentors, participants would initially engage in shallow or narrow discussion of particular issues in relation to how they affected access to abortion care and related SRH policies. However, over subsequent weeks, the discussions deepened, the debates became more engaging, the number of participants increased, and there quite some regular participants (of the trained health workers) who participated on the WhatsApp forum, call centre or in the bimonthly zoom continuous professional development (CPD) seminars. Considering that procuring abortion is judged negatively both morally and socially and that abortion on request is legally prohibited, there was limited sharing of experiences on provision of safe abortion at the beginning. Thus there was limited sharing and open discussions. This situation changed over the subsequent weeks, when the members knew more about each other, had built trust and were able to share freely. Thus, promoting trust, a sense of belonging and a supportive environment were key to successful establishment of the CoP.

The presence of facilitators/mentors (who were part of the initial trainer team) promoted sustainability of the CoP, since a facilitator plays a crucial role in addressing the challenges of establishing and nurturing a CoP [33, 34]. Facilitation can be defined as “making things easier by using a range of skills and methods to bring the best out in people as they work to achieve results in interactive events” [36, 37]. The facilitator role entails several behaviors, including directing, guiding, leading, counselling and giving feedback [38]. Prompting midwives to lead sessions and identify which topics to discuss was one way of promoting participation and leadership. In virtual environments, leadership ensures CoP members look to a facilitator to exercise leadership to a greater extent than in other kinds of virtual entities because COPs typically do not have an assigned leader. Good design requires an insider’s perspective of what the CoP is about. Facilitators are instrumental in helping a group capitalize on the technology’s potential to achieve meaningful interaction and learning.

Our study shows individual, interpersonal and organizational factors that influence success of a CoP. Individual-level factors such as previous experience, skills or social capital of an individual member, empowerment, motivation or confidence building are critical. Potential organizational mechanisms include nurturing trust between members and improving their level of communication and collaboration [37, 38]. System mechanisms include appreciation by the health supervisors, openness for (policy) changes and willingness to invest in time and resources for member participation [12–14]. The identified contextual factors include potential factors which can lead to influences at individual, interpersonal or organizational level. These include improved opportunities for knowledge acquisition or learning (individual outcomes), improved practice, sharing or implementation of new ideas (interpersonal and organizational outcomes), or quality improvements and policy changes (system outcomes) [12].

To promote success, the objectives and expectations of the CoP should be explicit to all those involved. The assumptions and expectations must be clarified to members, particularly the purpose of repeating some of the topics in the discussions, especially the need for moving progressively from theoretical to applied knowledge. The theory of change needs to be clear to all members to a CoP. To achieve CoP objectives, a more explicit ‘theory of change’, including how to monitor and evaluate effectiveness needs to be explicit. For our CoP, the assumption was that an initial competence-based training emphasizing knowledge, attitudes, skills and decision-making in using the AHR model, followed by both on-job mentorship and virtual mentorship (to reinforce sharing of knowledge and skills), regular communication and connection (to ensure sharing of experiences, challenges and concerns), as well as ongoing updates on clinical management, would foster a sense of community support and belonging, which would facilitate both rapid adoption of the AHR model and deeper learning through sharing, social learning and collaborative learning. Monitoring the CoP is key to assess whether activities are in line with a wider process of mobilization of resources for the achievement of explicit healthcare goals, as well as influencing health policy among stakeholders (such as clinicians, managers and analysts) [26]. As shown by our findings, the CoP strength lies in its promotion of an environment that is conducive to learning through knowledge sharing/exchange, by fostering social relationships and recognizing the importance of knowledge sharing through emphasis on interactions in a climate of mutual trust [2, 39, 40]. Our CoP was designed to strengthen adoption and implementation of the 2022 WHO guidelines on abortion care. Monitoring should entail decisions, plans, and actions undertaken to achieve specific healthcare goals and strategies to enhance exchange and co-production of knowledge [22, 38]. Thus, monitoring ensure that membership in the CoP enhanced both ability and willingness to initiate and use the AHR model, while addressing foreseen and emergent barriers to implementation.

Regarding opportunity, exposing trainees to new knowledge in a mode different from that in which it has traditionally been done (such as seminars and conferences) provided immense opportunity for learning and improvement in practice. While there is still clearly value in these forms of teaching and learning, the top/down approach, with internal or external ‘experts’ presenting to a relatively passive and unengaged audience has limitations in ensuring that either adequate knowledge is acquired or the acquired competences are implemented routinely during performance of the trainees. The respondents of the CoP found it easier to share, discuss and apply the knowledge shared from the different sessions. The sharing of views, values and experiences made it easier to reflective on personal and professional values and reduce value conflicts. Discussions also made it easier to grasp how new knowledge could be applied as well as how challenges encountered could be overcome, especially in relation to applying knowledge about the WHO 2022 abortion care guidelines, protocols for medical abortion and guidelines for instrument handling. Often, trainees and service providers identify growing dissatisfaction with this sort of professional development, conceived of as something that one ‘does’, or that is ‘provided’, or is ‘done to’ trainees [41]. However, some midwives often wanted topics such as obstetric complications included in the discussion, yet his was not part of the original plan. This is often a challenge [33]. Securing trust of shared information was also challenging. Lowering barriers among members to become involved in knowledge sharing activities was a challenge [33]. Besides, sustaining members’ active participation, since participation is central to the evolution of the community and to the creation of relationships that help develop the sense of trust, mutual sharing and collaboration [34–36].

### Perceived motivation

Healthcare providers are often isolated in their practice and individualism, rather than collaboration, is the norm. AOGU envisaged creation and supporting a CoP for SRH, specifically ending morbidity and mortality from abortion complications. CoPs, on their own or as part of larger interventions, may improve healthcare performance, and it was assumed that an opportunity to have for a for sharing updates, experiences, challenges and opportunities. A CoP represents a potentially valuable tool for creating, disseminating or sharing both tacit and explicit knowledge and implementation practices. The CoP may also be effective in creating links among the different ‘knowledge holders’ contributing to health practice or policy. From the midwife interactions, there were several benefits of creating CoP. They had potential to solve emerging problems especially stigma of abortion elf-care. The CoP enabled providers to explore ideas and act as sounding boards to each other. CoPs may create tools, standards, algorithms or job-aids. They also develop personal relationships and established ways of interacting, as well as a common sense of identity. The mentorship in the CoP promoted professional communication and understanding among and across different disciplines, cultures, perspectives, experience, languages. It encouraged technical exchange and professional development, providing benefits to both individuals and health facilities. For individuals, CoP participation increased access to technical resources, provide opportunities to contribute to discussions, and fostered a sense of membership and raised personal professional visibility. For organisations, the CoP allowed an effective way to raise awareness of SRH activities and services, and identified collaborators. CoPs were found to be less expensive, more interactive and more inclusive than physical conferences.

Though institutions are not set up for trainers to engage in “continuous and sustained learning”, the role of leadership in the CoP is critical in providing a regular, localised and supportive environment for engendering this sort of change in professional development cannot be ignored, and needs to be nurtured, embraced, supported and reinforced as the best way of ensuring that learning and training achieve the objective of improved provider performance [36, 37]. The CoP addresses the disparity between theory and practice and promotes sustainable service delivery suitable for different practical settings and contexts [5, 38]. Having an effective facilitator is key [10, 38–41, 43–45]. A facilitator leads a group towards achieving its objectives through designing and offering effective and efficient process structures, whether this takes place during a focused 2-h workshop or a multi-month period. Facilitators always provided a summary of learning points and take-home messages related to knowledge application. Most of the participants appreciated the idea of a CoP.

Lave and Wenger [39] emphasized that learning did not occur best from transmission of facts in the master/apprentice relationship, but rather, when facilitated within a community of apprentices and more experienced workers working together, interacting, sharing experiences and addressing concerns that arise through practice. Indeed, while CoPs were previously conceptualized as capable of emerging spontaneously in organizations, leadership and stewardship play a critical role in nurturing these communities [40] to ensure actualization of the three dimensions that define a CoP: joint enterprise (what it is about); mutual engagement (the interactions that lead to shared identity and meaning); a shared repertoire (of resources such as techniques, tools, experiences or process and practice) [41–45]. That COPs may bring different types of knowledge holders onto the same platform is very relevant because decisions on policies and their implementation are not only based on technical issues, but also on political and cultural as well as interactions between institutional actors and contextual factors [27, 43, 44]. In our context, these factors are exemplified by the role of personal and professional value conflicts which need to be addressed in implementation of the harm reduction model.

The concept of CoP as ‘groups of people who share a concern, a set of problems, or a passion about a topic and who deepen their knowledge and expertise in this area by interacting on an ongoing basis’ was coined by Wenger et al. [14] to describe a tool through which how novices (mentees) may learn from experts (mentors)within the contexts of workplaces. The CoP presents an innovative tool, where *“…new ideas are developed and implemented by people who engage in relationships with others and make adjustments needed to achieve desired outcomes within an institutional and organizational context”* [45]. Thus, learning in a CoP results from a social process consisting of knowing, acting, and structuring [44–46]. Nurturing trust, privacy and confidentiality while encouraging participation, communication and interaction is key for success in virtual communities of practice [33].

The primary benefits of a CoP is the individual practitioner largely by increasing their job efficacy through mutual leaning, social learning and collaborative learning [18]. This is manifest as people sharing knowledge through mutually guiding each other through their understandings of the same problems in their area of shared interest [18]. The learning value of a CoP stems from the groups collective intention and commitment to advance learning within the domain [47–50]. Over time, the shared history of learning also becomes a resource among the participants in the form of a shared repertoire of cases, techniques, tools, stories, concepts, and perspectives [39].

### Strengths and limitations

The study highlights technology, incentives, facilitation, leadership as key factors for success and sustainability of the CoP, thus ably presents the COP as a group of people who share a concern or a problem and who come together to connect, collaborate, cooperate, interact, learn and create a sense of identity, and in the process, build and share knowledge and solve problems. While the COM-B model elucidates on these factors ad their roles as barriers or facilitators of a CoP in the domains of capability, opportunity and organization, it may not delineate the organizational landscape or the deeper contextual factors in which the CoP is embedded, yet these may influence performance of the CoP, especially regarding effectiveness of the CoP. In outlining mainly the positive outcomes of the CoP, the model and the qualitative study design may not fully explain the potential challenges confronting CoPs, yet it may be such challenges that are crucial for performance and sustainability of the CoP. Outcomes are affected by context on different levels (individual, interpersonal or organizational), some of which are potentially modifiable by facilitators or members (such as structure, ways of interacting, nature of facilitation, shared resources or time) [47–49]. Since CoP structures allow members to draw on experience, reflect on action, and make adjustments after feedback on action [18, 49, 50], CoPs have the potential to drive systems change. In this way, CoPs can interrogate basic assumptions that underlie current policies, practices, and programs with the aim of system improvements [49, 50]. In our context, the CoP was able to adopt both the AHR and VCAT for improving access to abortion care.

## Conclusions

This case study presents data on initiation and operation of a CoP among health workers, with related challenges in both conceptualization and implementation. The CoP was able to facilitate healthcare providers to take collective responsibility for managing the knowledge they needed, create a connection between learning and performance, address knowledge creation and sharing, and create connections and interactions among themselves. The CoP created social space in which members furthered learning. The domain was increased access to CAC using VCAT and the abortion harm reduction model. The community members were doctors and midwives, while the practice was increased access to abortion care using the harm reduction model. The findings confirm that a CoP is not merely a network of connections between people, but has an identity defined by a shared domain of interest, type of members, their interaction and the cultural context in which the CoP is embedded. The starting point is its domain- what initially motivates people to gather, with a shared concern or interest. In pursuing their interest in their domain, members should engage in joint activities and discussions, help each other, and share information. They should build relationships that enable them to learn from each other; they care about their standing with each other. The dynamic nature of the CoP is key to their evolution and effectiveness.

## Data Availability

Not applicable.

## Abbreviations

AHR: Abortion harm reduction
AOGU: Association of Obstetricians and Gynecologists of Uganda
APMM: Advocacy for prevention of morbidity and mortality
CAC: Comprehensive abortion care
CoP: Community of Practice
PAC: Postabortion care
SRH: Sexual and Reproductive Health
SRHR: Sexual and Reproductive Health and Rights
VCAT: Value clarification and attitude transformation
